# Case report: Radical robotic nephroureterectomy after chemotherapy followed by avelumab in a patient with node-positive UTUC

**DOI:** 10.3389/fonc.2024.1465213

**Published:** 2024-11-11

**Authors:** Hana Studentova, Vladimir Student, Daniela Kurfurstova, Andrea Kopova, Bohuslav Melichar

**Affiliations:** ^1^ Department of Oncology, Faculty of Medicine and Dentistry, Palacky University and University Hospital, Olomouc, Czechia; ^2^ Department of Urology, Faculty of Medicine and Dentistry, Palacky University and University Hospital, Olomouc, Czechia; ^3^ Department of Clinical and Molecular Pathology, Faculty of Medicine and Dentistry, Palacky University and University Hospital, Olomouc, Czechia

**Keywords:** upper urinary tract carcinoma, UTUC, neoadjuvant chemotherapy, radical nephroureterectomy, immunotherapy, immune checkpoint inhibitors, cancer-specific survival

## Abstract

**Introduction:**

Platinum-based chemotherapy followed by the immune checkpoint inhibitor avelumab represents an intensified upfront therapy regimen that may result in significant downstaging and, subsequently, potentially radical robotic nephroureterectomy with a lymph node dissection, an uncommon approach with an unexpectedly favorable outcome.

**Case presentation:**

We report a case of a 70-year-old female presented with a sizeable cN2+ tumor of the left renal pelvis and achieved deep partial radiologic response after systemic therapy with four cycles of gemcitabine-cisplatin chemotherapy followed by avelumab maintenance therapy and subsequent robotic resection of the tumor. The patient continued with adjuvant nivolumab therapy once recovered after surgery and remained tumor-free on the subsequent follow-up. The systemic treatment was without any severe adverse reaction.

**Conclusion:**

We highlight the feasibility of the upfront systemic therapy with four cycles of gemcitabine-cisplatin chemotherapy followed by avelumab maintenance, robotic-assisted removal of the tumor, and adjuvant immunotherapy with nivolumab. This intensification of the upfront systemic therapy, and the actual treatment sequence significantly increase the chances of prolonged survival or even a cure. This type of personalized therapeutic approach can accelerate future advanced immunotherapeutic strategies.

## 1 Introduction

The upper tract urothelial carcinoma (UTUC) is a rare but aggressive tumor in which timely and appropriate diagnosis is crucial. Compared to bladder cancer, UTUC is often diagnosed at an advanced stage and is associated with a worse prognosis than urothelial carcinoma (UC) of the lower urinary tract, with 5-year cancer-specific survival (CSS) rates of 57-73% (1, 2). The standard therapeutic approach is radical nephroureterectomy (RNU) with a template lymph node dissection (LND) followed by adjuvant chemotherapy in indicated cases based on the POUT trial (3, 4). However, the prognosis remains poor, and specific clinical characteristics of individual patients should be considered when determining the optimal therapeutic strategy based on the risk stratification of these tumors. The neoadjuvant approach in muscle-invasive bladder cancer (MIBC) has become the mainstay of the therapy (5). Since UTUC is an entity of different origins and biology, the guidance on treatment cannot be derived from MIBC. In the meantime, the level of evidence supporting the neoadjuvant approach for UTUC is relatively low. Neoadjuvant chemotherapy (NAC) confers a favorable outcome in terms of pathologic complete response (pCR) and downstaging rate, leading to improved overall survival (OS) benefits compared with RNU alone, but this is supported only by retrospective data (6–11). Meanwhile, recurrences and high mortality rates in the long term are common. The guidance of the European Association of Urology on using NAC in UTUC remains cautious, and no firm recommendation can be made due to the absence of randomized clinical trials and inconclusive findings from meta-analysis (12). There is no prospective evidence regarding the management of clinical node-positive disease. Patients with node-positive UTUC should be offered systemic first-line platinum-based chemotherapy followed by avelumab maintenance if the response to chemotherapy is obtained (13). Surgical resection, including LND, should be discussed within a multidisciplinary team in patients responding to systemic therapy whenever feasible (14).

In recent years, immune checkpoint inhibitors (ICIs) have entered the UC therapeutic landscape. Undoubtedly, the immunotherapeutic approach significantly increases the chance of long-term disease control. The experience with ICIs across various tumor types has shown unparalleled advances in the treatment, resulting in cancer cell elimination. However, more progress has yet to be made in identifying patients who would benefit from an immune-targeted approach. Regarding ICIs in high-risk UTUC, there is hardly any evidence for immunotherapy in high-risk UTUC patients in preoperative settings. It is only adjuvant nivolumab in muscle-invasive UC with tumor cell PD-L1 expression >1% and a high risk of recurrence following the surgery considered a standard of care based on the Checkmate-274 trial, including patients with UTUC. However, no benefit was described in this patient subgroup (15). Meanwhile, adjuvant platinum-based chemotherapy is superior to ICIs in this patient population (16).

We describe here a patient with locally advanced inoperable UTUC treated with initial cisplatin-based chemotherapy followed by three months of avelumab. This led to significant downstaging of the disease, resulting in robotically assisted resection of the primary tumor concurrently with LND 8 months later. Adjuvant immunotherapy with anti-PD1 antibody was initiated to secure long-term disease control.

## 2 Case presentation

**Figure 1:**
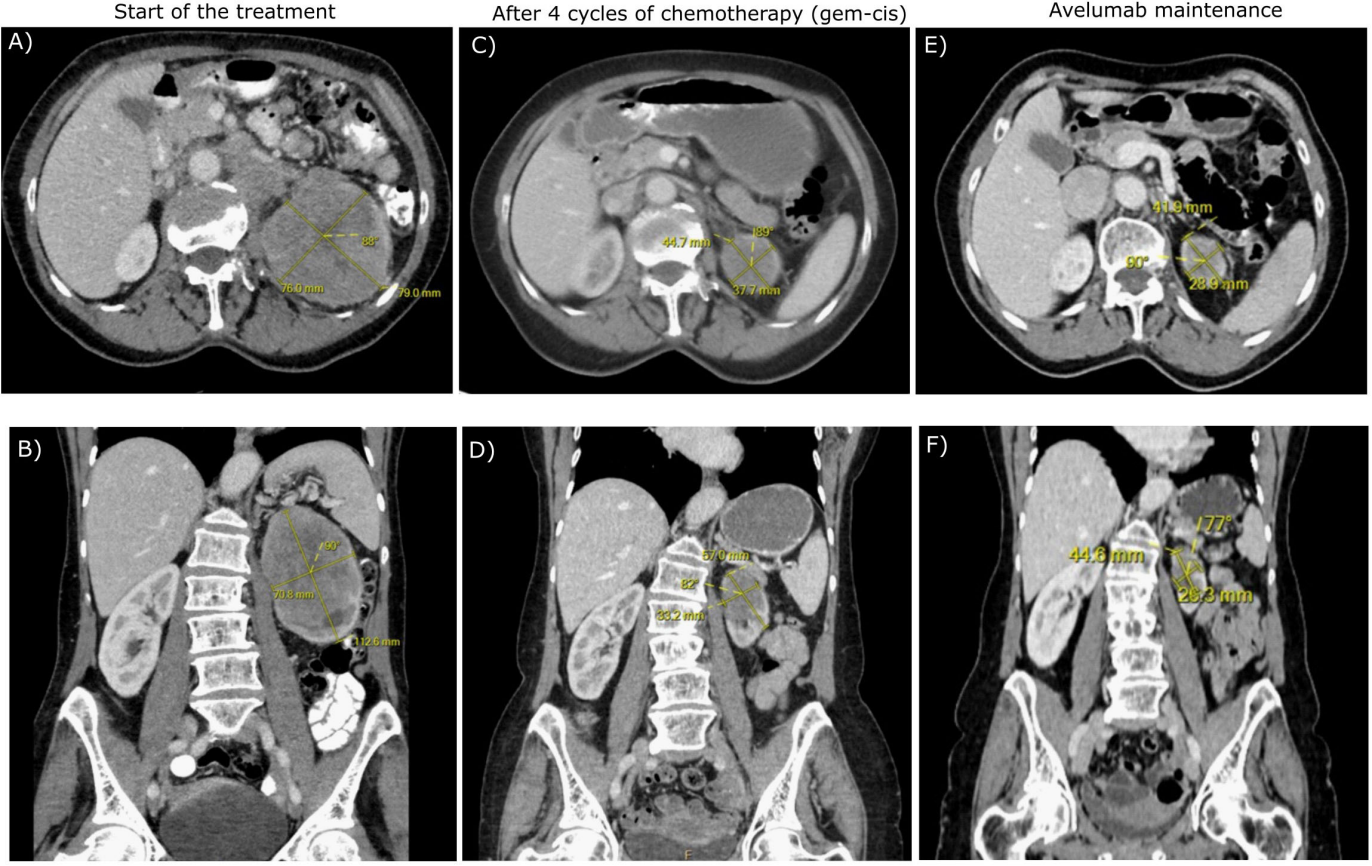
The transverse **(A, C, E)** and frontal **(B, D, F)** CT scans of the patient during the treatment.

**Figure 2:**
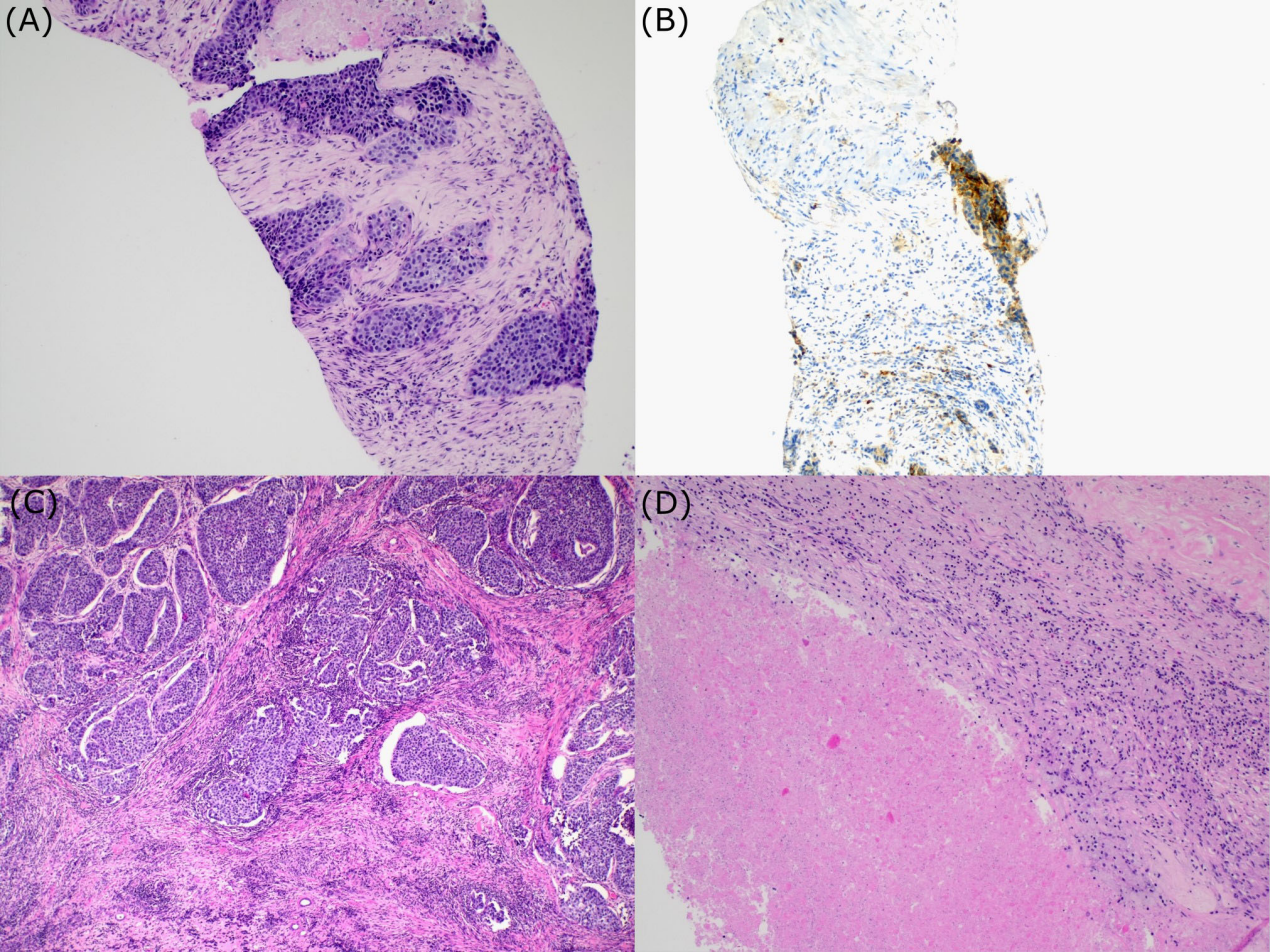
Histologic examination. Bioptic sample of urothelial carcinoma **(A)**; PD-L1 expression **(B)**; Postsurgical specimen. Section showing areas of urothelial carcinoma surrounded by fibrosis and chronic inflammation **(C)**. Postsurgical specimen. Regions of necrosis within the tumor **(D)**.

A 70-year-old Caucasian female, light smoker, with an Eastern Cooperative Oncology Group (ECOG) status 0 and no severe comorbidities, presented in April 2023 with flank pain and hematuria. The abdominal computed tomography (CT) scan revealed a large tumor of the left kidney with bulky retroperitoneal lymphadenopathy surrounding the large abdominal vessels. The tumor dimensions were 79x 76 x 112 mm (**Figures 1A, B**). The chest CT and bone scan showed no evidence of metastatic disease, the tumor stage cT3cN2. An ultrasound-navigated tumor biopsy was performed, and a histological examination revealed high-grade UC with a PD-L1 expression of 15 (**Figure 2**), according to a combined positive score (CPS). The laboratory tests showed an elevated CRP level of 53.4 mg/l, anemia (103 g/l), thrombocytosis (459 x 10^9^/l), neutrophilia (7.25 x 10^9^/l) and CYFRA 21-1 46.57 ug/l. Upon review of imaging during the multidisciplinary board, the tumor was deemed unresectable due to the size, lymph node involvement, and proximity to the renal vasculature, also taking into consideration apparent aggressive biology. After an in-depth discussion with the patient, surgical resection was not deferred, and the patient opted to proceed with systemic chemotherapy.

**Figure 3:**
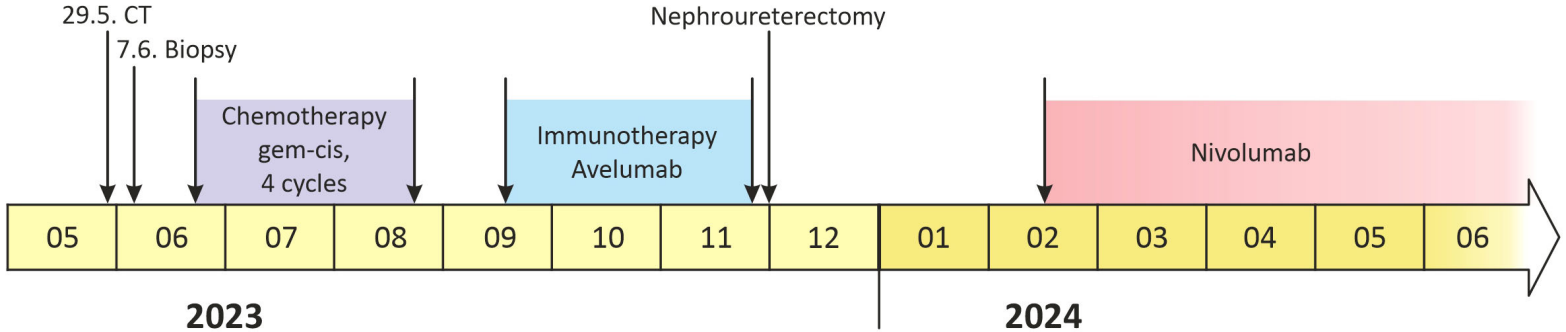
Timeline of the treatment.

Chemotherapy with cisplatin (75mg/m^2^) and gemcitabine (1000mg/m^2^) was initiated in June 2023. In August 2023, the restaging CT scan was performed, demonstrating tumor size reduction to 37 x 44 x 57 mm; i.e. partial regression by Response Evaluation Criteria in Solid Tumors (RECIST) 1.1 (**Figures 1C, D**). After four cycles of chemotherapy, avelumab maintenance therapy was started. The patient tolerated the treatment well with no side effects. In September 2023, the CT scan showed an additional tumor size decrease with no evidence of distant metastases (**Figures 1E, F**). The patient continued with avelumab monotherapy, and the PET/CT with ^18^F-fluordeoxyglucose confirmed ongoing partial response. The multidisciplinary tumor board team decided to proceed with radical tumor resection. In December 2023, after six cycles of avelumab, radical robotic-assisted resection, including left nephroureterectomy (RRNU) and template LND, was performed successfully with an uneventful postoperative course, although LND could not have been radical due to proximity to large vessels. Final histological analysis showed an end-stage kidney with residual high-grade UC (**Figure 2D**); pathologic TNM classification was ypT1a ypN0 with negative margins, and angioinvasion was evaluated as a significant partial response. The timeline of the treatment is summarized in **Figure 3**.

In January 2024, adjuvant nivolumab was initiated due to the uncertainty regarding persistent lymph nodes in the retroperitoneum. Fourteen months after the initial diagnosis, the patient remains asymptomatic and disease-free on CT. During therapy with nivolumab, the patient developed hypothyreosis that was supplemented with levothyroxine.

## 3 Discussion

We report here an excellent outcome of cisplatin-based chemotherapy followed by avelumab maintenance given in a preoperative setting in a patient with cT3cN2 UTUC. The therapy resulted in significant tumor shrinkage and subsequent successful robotic-assisted surgical resection of the tumor along with LND. The patient was offered adjuvant immunotherapy for one year in an effort to obtain durable remission. To the best of our knowledge, this may be the first report where the downsizing of the primary unresectable tumor, achieved by cisplatin-based chemotherapy followed by avelumab, led to radical tumor resection in patients with UTUC.

Neoadjuvant therapy in UTUC remains under investigation, and no systemic therapy is currently considered a standard of care in localized or locally advanced diseases. It is crucial to downstage the tumor, potentially making the surgical resection feasible while improving the outcome. Because it is given preoperatively in patients with potentially the best renal function possible, NAC is an obvious advantage compared to adjuvant chemotherapy. Although there are no prospective randomized data, two phase II prospective clinical trials demonstrated that NAC based on cisplatin and gemcitabine combination in high-risk UTUC results in a pathologic complete response pCR rate of 14-19% (17, 18). Evidence from systematic reviews and meta-analyses has been inconclusive, given the significant bias and heterogeneity (19, 20). Systematic review and meta-analysis by Leow et al. showed pCR in 43% and clinically significant downstaging in 33% of high-risk UTUC patients following NAC, resulting in improved OS and cancer-specific survival compared with RNU alone (21). Recently, a meta-analysis by Deb et al. reported a 10% pCR rate in UTUC patients receiving NAC (22). According to their analysis, nearly half of the NAC patients achieve a significant reduction in tumor mass, facilitating the surgical intervention and demonstrating a substantial decrease in the risk of death at five years in the post-NAC group. On the contrary, the survival benefit decreases over time, showing no significant difference in the long term, which is in concordance with previously published studies (19, 23). The meta-analysis also revealed a 31% rate of advanced disease (pT3-pT4), i.e. a significantly lower risk of advanced disease in patients receiving NAC, again suggesting the crucial role of NAC in UTUC patients. Additionally, patients with residual invasive UC following NAC have poor prognosis, and optimal treatment strategy should be determined to improve the outcome in the setting (24).

However, the studies show substantial heterogeneity and the extent of variability in response of significant importance, necessitating a tailored approach with individualized patient assessment and demand for reliable predictive biomarkers. Patient stratification relies on clinical examination, imaging, histology, and the risk assessment of Lynch syndrome. Several prognostic factors have been identified based on retrospective trials, including preoperative nutritional status, tumor stage, tumor grade, and other tumor-related prognostic factors, allowing patient stratification and better selection when perioperative management is considered (25–29). Several blood-based biomarkers have been investigated in patients with UTUC regarding survival prediction. Mori et al. published a meta-analysis reporting that differences in CRP, neutrophil-to-lymphocyte ratio, white blood cells, hemoglobin, and estimated glomerular filtration rate are associated with CSS (30).

In the presented case, due to the sizeable inoperable tumor mass and bulky lymph nodes, we decided to initiate cisplatin-based chemotherapy and continued with avelumab maintenance immunotherapy. The therapy was successful in downsizing the tumor, but the principal concern was the limited effect of chemotherapy, which we attempted to overcome by adding avelumab. Failure to maintain disease control adversely affects the patient’s prognosis. Based on real-world clinical experience and available reports, there is a long-term concern that the initial success of NAC is not durable and potential disease recurrence may often be inevitable (19, 23). Studies reporting results of NAC in patients with UTUC are summarized in **Table 1**. NAC can influence only short-term prognosis, not persisting over extended periods. The present case report shows the efficacy of preoperative chemotherapy in UTUC patients, allowing for surgery in initially very advanced diseases. Disease recurrence is common in the setting, and incorporating ICIs in the perioperative strategy could increase the chance of disease control. However, there is no persuasive data on using ICIs preoperatively and only two trials have been published. The PURE-02 trial evaluated 10 patients treated with pembrolizumab in the neoadjuvant setting with no pCR (31). Another phase II study in cisplatin-ineligible UTUC patients investigated the efficacy of ipilimumab and nivolumab given preoperatively. Three out of 9 patients achieved pCR, while the remaining had downstaging. Next-generation sequencing revealed germline variants in mismatch repair genes in all three patients with pCR (32). In the preoperative setting, only a few sporadic cases of immunotherapy or combination with chemotherapy were published using different drug combinations with a short follow-up (33, 34). Ikarashi et al. published an extraordinary case of pCR achieved by pembrolizumab in a preoperative setting in a patient with UTUC progressing on NAC (35). An overview of case reports in UTUC patients treated with perioperative immunotherapy is shown in **Table 2**.

**Table 1:** Studies reporting results of neoadjuvant chemotherapy in patients with UTUC.

Publication	Study type (Year of publication)	Number of patients (NAC – neoadjuvant chemotherapy)	Results
Matin (6)	Retrospective single center (2010)	43 NAC MVAC, CGI, GTA, GC vs. 107 (control without NAC)	14% CR in NAC vs. none in the control group
Porten (51)	Retrospective single center (2014)	31 NAC (cisplatin-based and other) vs. 81 (control without NAC)	NAC: better disease-specific survival HR 0.19 (0.06-0.61)
Kobayashi (52)	Retrospective single center (2016)	24 NAC (cisplatin-based) vs. 31 (control without NAC)	NAC: better OS HR 0.47 (0.22 -0.99)
Kubota (53)	Retrospective multicenter (2017)	101 NAC gemcitabine-carboplatin (75%) or gemcitabine-cisplatin (21%) vs. 133 (control without NAC)	25% downstaging vs. 14% downstaging NAC: better CSS HR 0.48 (0.26-0.87)
Hosogoe (54)	Retrospective single center (2018)	55 NAC gemcitabine-carboplatin (100%) vs. 138 (control without NAC)	63% downstaging vs. 24% downstaging NAC: better CSS HR 0.37 (0.15-2.62)
Zennami (55)	Retrospective single center (2021)	117 NAC gemcitabine-carboplatin, MVAC vs. 67 (control group without NAC)	NAC: better OS HR 0.417 (0.231–0.754)
Kohada (56)	Retrospective single center (2023)	20 NAC (gemcitabin – cisplatin) vs. 24 (control group without NAC)	NAC: better OS in renal pelvis tumor cohort HR 0.15 (0.03-0.66)
Coleman (18)	Multicenter single-arm, phase II trial (2023)	57 NAC (gemcitabine – cisplatin)	19% CR 63% pathological response

MVAC, methotrexate, vinblastine, doxorubicin, and cisplatin; CGI, cisplatin, gemcitabine, and ifosfamide; GTA, gemcitabine, paclitaxel, and doxorubicin; GC, cisplatin and gemcitabine; NAC, neoadjuvant chemotherapy; HR, hazard ratio; OS, overall survival; CR, complete response; CSS, cancer-specific survival.

**Table 2:** UTUC case reports with perioperative immunotherapy.

Publication	Care report	1. step + outcome	2. step + outcome	3. step	Outcome
Ikarashi (35)	57 y male Heavy smoker T3N0M0 UTUC PD-L1 not known at the start of NAC	I. line NAC Gemcitabine-Carboplatin (2 cycles) Tumor enlargement	II. line Pembrolizumab (5 courses) 90% shrinkage	Nephroureterectomy + Lymphadenectomy **Tumor PD-L1 positive**	No local recurrence or metastasis after 1 year at the time
Chan (34)	81 y female Hypertension T3N0M UTUC bilateral **High expression of** **PD-L1 (60%)**	I. line NAC Pebrolizumab (3 cycles) right pelvis CR left pelvis PC	Nephroureterectomy Left pelvis	Additional Pebrolizumab (2 cycles)	No local recurrence or metastasis after 3 year at the time
Ni (57)	71 y male UTUC **PD-L1 (6%)**	Nephroureterectomy	I. line Gemcitabine-cisplatin (6 cycles) Multiple metastasis	II. line Camrelizumab (8 cycles) PR	PFS 5 months
Xu (33)	66 y male cT3-4N1M0 UTUC left renal pelvis **PD-L1 (TPS 10%)**	I. line NAC Tislelizumab + Gemcitabine-Carboplatin (4 cycles) PR	Nephroureterectomy + Lymphadenectomy	Adjuvant therapy Gemcitabine-Carboplatin (4 cycles)	No signs of the carcinoma returning
Chan (58)	84 y female T3N1Mx UTUC left renal pelvis PD-L1 not known FGFR3, PIK3CA, BRCA1, BRCA2	I. line NAC Pembrolizumab- Enfortumab Vedotin	Radical nephroureterectomy	–	CR

CR, complete response; PR, partial response; PFS, progression-free survival. The PD-L1 expression is highlighted in bold font.

In the presented case, the tumor expressed PD-L1 with a CPS of 15. PD-L1 positivity was previously described as an independent prognostic factor with a favorable OS outcome in UTUC patients with organ-confined disease (36). On the contrary, luminal subtype UC enrichment is often seen in UTUC characterized by so-called “immune-dessert” predicting resistance to pembrolizumab, as previously reported (37, 38). Long-term control was attempted by introducing immunotherapy in the preoperative setting using an anti-PD-L1 antibody, avelumab, as the only ICI registered as maintenance following first-line platinum-base chemotherapy. After recovery from the surgery, we opted for adjuvant nivolumab as the only molecule that proved efficacy in high-grade muscle-invasive UC in the postoperative setting. Potential antitumor response promoted by ICIs via PD-1/PD-L1 inhibition may induce a long-term effect by metastatic clone elimination.

RNU represents a challenging procedure that includes ablative (nephrectomy, urethrectomy, and lymphadenectomy) and reconstructive (bladder cuff excision and bladder reconstruction) components. Tumor staging is inaccurate preoperatively; hence, regarding the extent of lymphadenectomy, template-based LND should be the preferred option, having a better impact on patient survival than the number of lymph nodes removed (39). The robotic-assisted nephroureterectomy (RRNU) is emerging as a new gold standard for RNU (40). Although laparoscopic RNU shows better perioperative outcomes than open RNU, it is flawed because of worse oncological outcomes, especially in non-organ-confined diseases (41, 42). Comparative studies suggest similar oncological outcomes of the RRNU with open and laparoscopic surgery while showing lower blood loss, shorter hospitalization stays, and fewer complications in RRNU (43–45). RRNU is increasingly used in the US (46) and Europe (47), exceeding the share to more than 50% of cases. This is also true for cT3-4 and cN+ disease (47), where the guidelines give a weak recommendation to perform an open approach (12). Studies show higher utilization of LND in RRNU compared to open and laparoscopic techniques, which leads to improved nodal yield and possibly can improve the survival rates in RNU (43, 48, 49).

In the presented case, no serious complications apart from hypothyreosis were associated with systemic therapy, surgery, or wound healing. The safety and tolerability of a multidisciplinary approach are paramount in UTUC management, considering the complexity and potential risks arising from given systemic treatment and major surgical procedures. Wound healing problems resulting from NAC have been previously described, as were significant fibrotic changes induced by immunotherapy and encountered during the surgery, leading to increased postoperative morbidity (50). A sequence of both approaches may become highly demanding for the operating surgeons. Nevertheless, NAC is generally considered a safe and well-tolerated approach at UTUC.

## 4 Conclusion

The present case report demonstrates the potential of cisplatin-based chemotherapy followed by avelumab in UTUC in a preoperative setting to downsize an initially inoperable lymph node-positive UTUC, leading to potentially radical surgical resection. Nivolumab, given as adjuvant treatment, was attempted to increase the chance of durable remission. While longer follow-up is awaited for the definitive cure to be secured, the current case report justifies the sustained support of trials using this strategy. The personalized multidisciplinary approach can bring up new potential strategies for future clinical translation.

## Data Availability

The original contributions presented in the study are included in the article/supplementary material. Further inquiries can be directed to the corresponding author.
